# Biomechanical effects of Skeletally anchored Class III elastics on the maxillofacial complex: a 3D finite element analysis

**DOI:** 10.1186/s40510-021-00375-3

**Published:** 2021-10-25

**Authors:** Priyank Rai, Dhiraj Garg, Tulika Tripathi, Anup Kanase, Gayatri Ganesh

**Affiliations:** Department of Orthodontics and Dentofacial Orthopaedics, Maulana Azad Institute of Dental Sciences, Bahadur Shah Zafar Marg, New Delhi, 110002 India

## Abstract

**Background:**

Although, the outcomes and changes in the maxillofacial complex after the application of intraoral bone anchored Class III elastics, have been reported by multiple clinical studies, there was no finite element study to assess and evaluate the stress pattern and displacement on maxillomandibular complex with bimaxillary anchorage. The present study aims to evaluate the biomechanical effects on maxillomandibular complex of Skeletally anchored Class III elastics with varying angulations using the 3D finite element analysis.

**Methodology:**

Two 3-dimensional analytical models were developed using the Mimics 8.11 (Materialise: Leuven, Belgium) and ANSYS software Version 12.1 (ANSYS Inc, Canonsburg, PA, USA) from sequential computed tomography images taken from a Skeletal Class III subject. The models were meshed into 465,091 tetrahedral elements and 101,247 nodes. Intraoral mechanics for skeletally anchored maxillary protraction (I-SAMP) were applied on two models i.e. A and B (without and with maxillary expansion respectively) between miniplates on maxilla and mandible on both right and left sides with three different angulations of forces—10°, 20° and 30°).

**Results:**

Although the craniomaxillary complex in both the models (A and B) displaced forward while demonstrating rotations in opposite directions, the displacements and rotations decreased gradually with the increase of the angle of load application from 10° to 30°. The mandible rotated clockwise in both the simulations, but the displacement of mandibular surface landmarks was higher in Simulation A. However, the antero-inferior displacement of the glenoid fossa was higher in Simulation B than in A.

**Conclusion:**

Significant displacement of maxillofacial sutures and structures was witnessed with I-SAMP with maxillary expansion and Class III elastics for correction of Skeletal Class III with maxillary retrognathism. Thus, I-SAMP with maxillary expansion is a desired protocol for treatment of maxillary retrognathism. However, the prescribed angulation of the Class III elastics should be as low as possible to maximise the desired effects.

## Introduction

Skeletal Class III malocclusion is considered to be one of the most formidable malocclusions to treat in orthodontics. The prevalence rate of this malocclusion varies between 1 and 2% in the Indian population and 0–26% worldwide [1]. Features commonly associated with this malocclusion include a concave profile, retrusive paranasal region, protruded lower lips and a prominent chin [2]. The treatment modalities for Skeletal Class III are often governed by a number of factors such as the degree of skeletal malocclusion, age of the individual, etc.. Early presentation of Skeletal Class III is usually corrected by using a conventional facemask, functional regulator-III, reverse twin block or a chin-cup [3]. On the other hand, treatment in adults warrants correction by orthognathic surgery. In adolescents, the treatment options become limited due to the patient’s disagreement with extra oral anchorage sources or existing morphological limitations. The introduction of skeletal anchorage for Skeletal Class III correction [4, 5] solved both the above purposes while maximising the skeletal effects and reducing the undesirable dental effects [6, 7].

Maxillary protraction by conventional methods usually results in the forward and downward movement of the maxilla and the maxillary dentition, while the mandible and its dentition move downward and backward [8]. Protraction of the maxilla may be accompanied by RME (rapid maxillary expansion) leading to the forward displacement of the entire maxillary complex and the anterior morphogenetic rotation of the mandible due to the significant forward and upward growth of the mandibular condyle [9]. The application of orthopaedic forces generates stresses and strains on the entire maxillomandibular complex which can be evaluated non clinically using the finite element method (FEM) [10].

The finite element method is a computer-aided numerical technique for finding approximate solutions to physical systems subjected to external influences under specific boundary conditions in order to solve complex problems in engineering, science and applied mathematics [11]. The application of FEM to study maxillary protraction using intra-oral skeletally anchored miniplates only in the maxilla has been done by researchers such as Lee et al. [12] and Moon et al., [13] who have found that by varying the location and vector of Class III mechanics, orthodontists can differentially alter the magnitude of forward, downward and rotational movements of the maxilla.

Although multiple clinical studies elaborating the outcomes and changes in the maxillofacial complex after application of intra-oral bone-anchored Class III elastics have been reported [4, 14], there was no finite element study to assess and evaluate the stress pattern and displacement on maxillomandibular complex with bimaxillary anchorage. Thus, the present study aims to evaluate the biomechanical effects on maxillomandibular complex of Skeletally anchored Class III elastics with varying angulations using the 3D finite element analysis.

## Materials and methodology

The present study was approved by the Institutional ethical committee (MAIDS/2016). A CBCT scan of the maxillofacial complex of a 10-year-old female with a retrusive maxilla, protrusive mandible and an anterior crossbite was used for the FEM study. The informed assent was obtained from the patient and her parents. The patient was diagnosed and subsequently treated in the department.

### Preprocessing stage

**Fig. 1:**
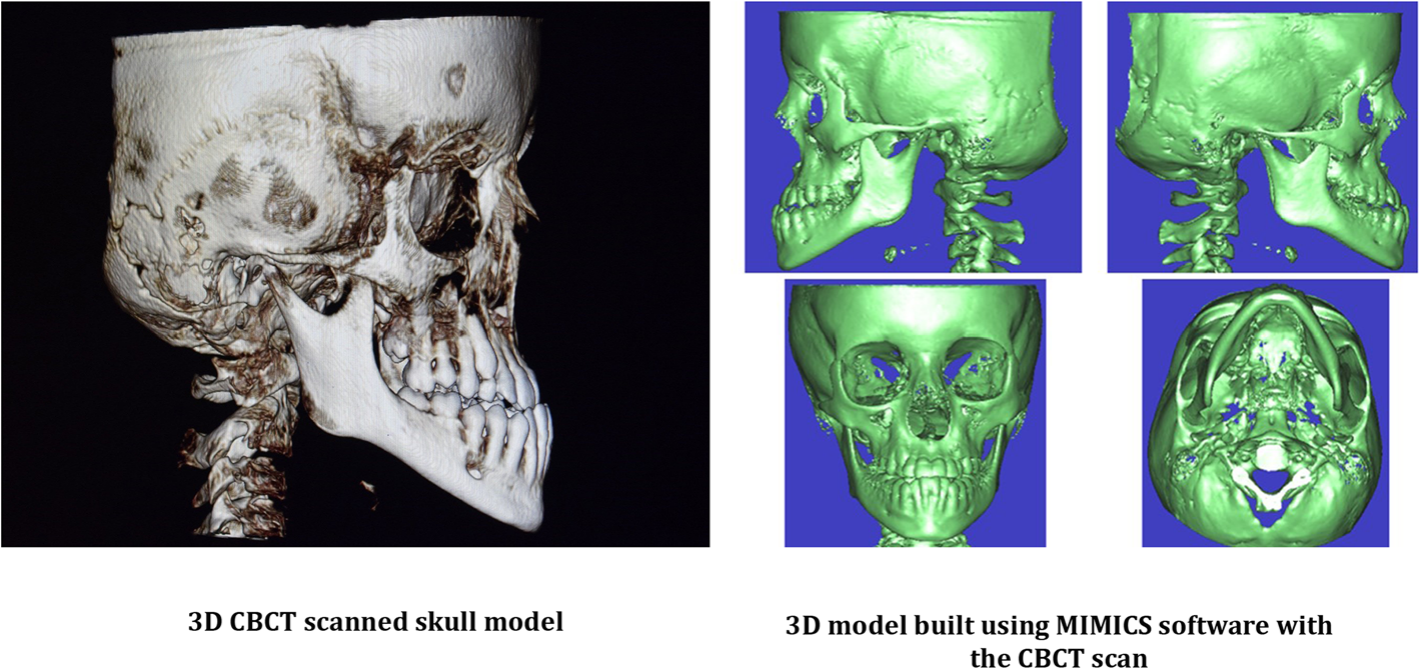
3D CBCT scanned skull model and 3D model of maxillofacial bones built by the Mimics software after the CT scan

The raw volumetric DICOM data from the CBCT scan was imported into a finite element modelling software package, Mimics 8.11 software (Materialise: Leuven, Belgium), and a 3D model of the patient's skull was generated as shown in Fig. 1. The 3D mask of the skull was then modified to create separate masks for the sutures, bones and teeth.

**Fig. 2:**
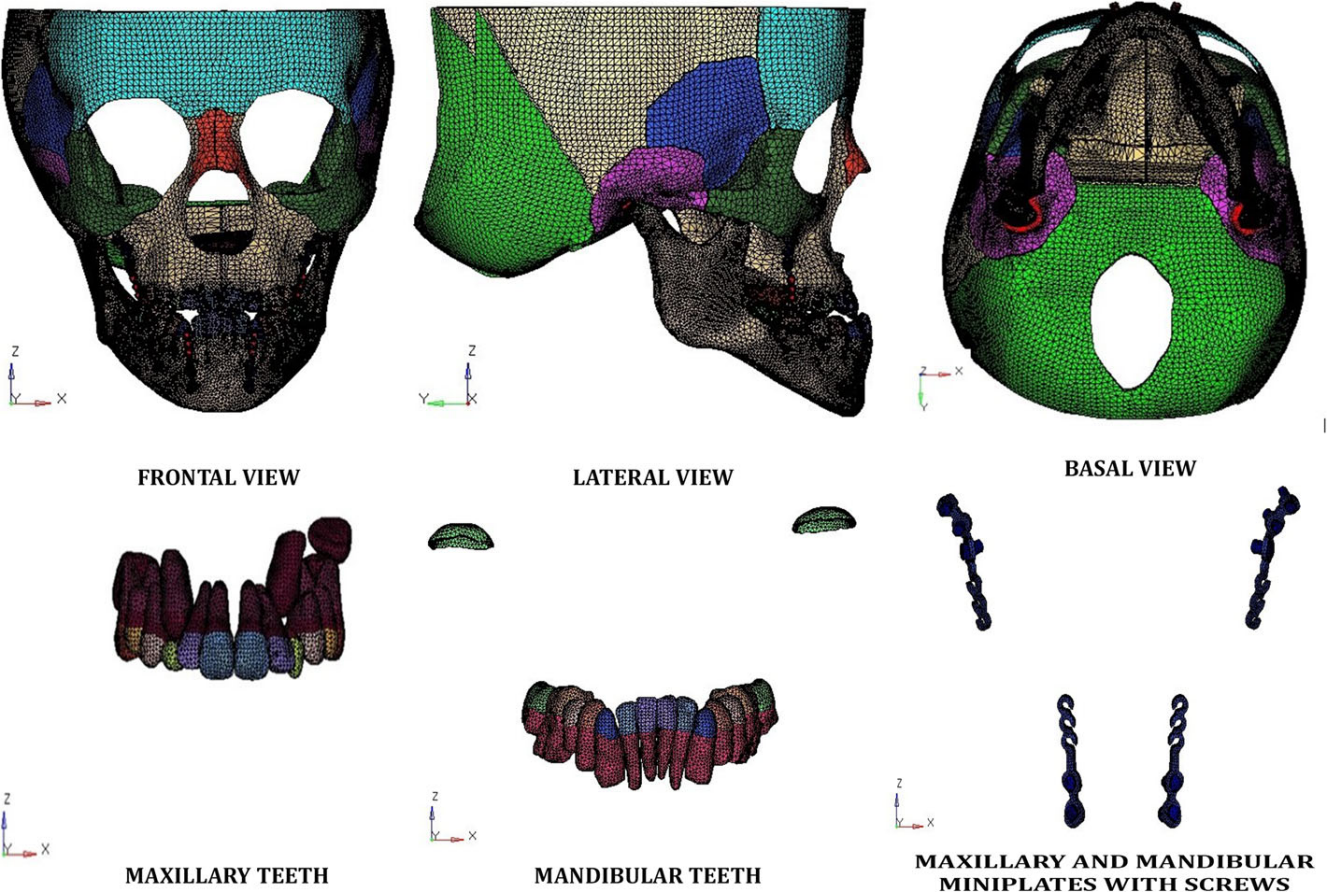
Three-dimensional mesh FE model with the maxillary, mandibular teeth and miniplates and screws

Nine craniofacial sutural systems namely the Frontonasal, Frontomaxillary, Zygomaticomaxillary, Zygomaticotemporal, Zygomaticofrontal, Pterygomaxillary sutures, Internasal, Nasomaxillary and Sphenozygomatic were integrated into the model by identifying and duplicating nodes corresponding to the anatomic sutures. Multiple nodes were created along the entire suture length with the thickness of each suture modelled at 0.8 mm. The mathematical model subsequently generated did not assume any mobility in the sutures, so that the strain values were determined only by the material and geometric properties of the skull. The reconstructed geometry of craniomaxillary complex was exported in Stereolithography (STL) file format as the geometric model. This geometric model in (STL format) was further imported into the Hypermesh Version 13.0 (Altair Computing, Inc, Troy, MI, USA) and subsequently meshed into 465,091 tetrahedral elements and 101,247 nodes. The materials used in the discretized model were assumed to be isotropic, homogenous and linearly elastic based on previous studies [12, 15, 16] (Fig. 2).

### 3D FEM models for the maxillary and mandibular miniplates (Fig. 2)

A 3D finite element model for a curvilinear type of surgical miniplate with 3 (upper) / 2 (Lower) holes and 3 hooks (Table 1) was designed based on 3D computer-aided design data and fixed according to the anatomic shape of the infrazygomatic crest in the maxilla and in between canine and lateral incisor on the mandible by the projection method. In the mandibular arch, the distal end of a similar miniplate was placed at the occlusal level between the maxillary canine and the lateral incisor and the mesial end of the miniplate was located near the lower border of the mandible.

**Table 1:** Characteristics of the surgical miniplate (S. K. Surgicals, Pune, Maharashtra, India)

	Measurement
Thickness	0.80 mm
Length	Upper (31.65 mm) & lower (21 mm)
Hole diameter	2 mm
Distance between the centres of holes	5.50 mm
Curvature	0.04 mm

### Solution stage

Force loading of the miniplates was done by applying protraction forces (250 g/side) between different hooks of both the maxillary and mandibular miniplates bilaterally and two 3-dimensional analytic models were developed namely A and B. Simulation A simulated maxillary protraction alone with 250 g of force applied in between different hooks on the distal ends of both maxillary and mandibular miniplates bilaterally. Simulation B included a maxillary protraction with expansion, where a transversal (X) displacement of 2 mm was applied on the surface nodes of the intermaxillary suture on both sides in the first molar region to simulate the initial phase of maxillary expansion. It was assumed that the two plates of the transversal orthopaedic appliance moved apart by a total distance of 4 mm after which the maxillary protraction forces of 250 g were applied as in Simulation A.

Three different clinical protocols for maxillary protraction were simulated by varying the location and direction of the forces applied.

**Fig. 3:**
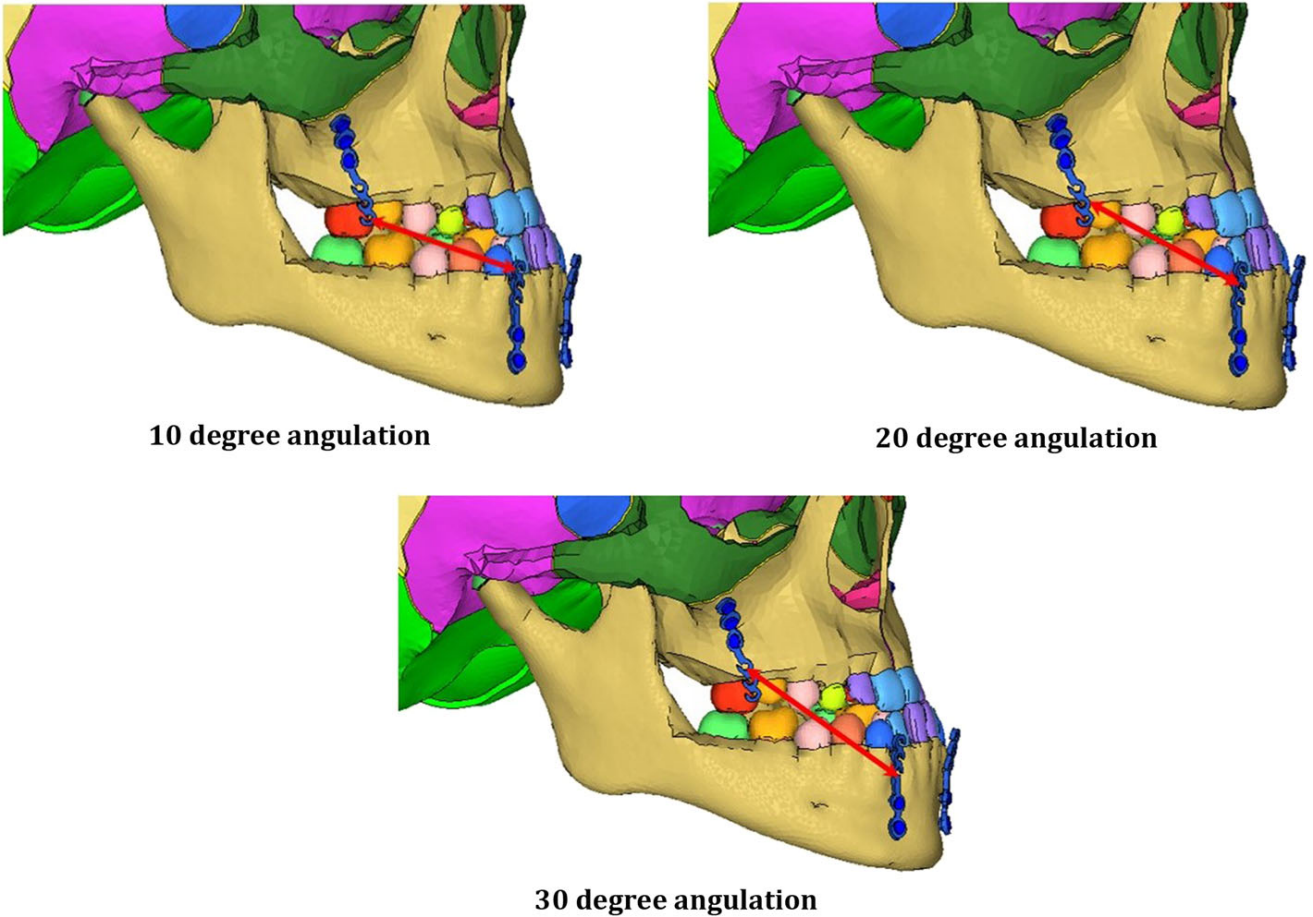
Finite element analysis with the three angulations of elastics (10° to 30°)

1. *In the 10° angulation model*, 250 g of load connects distal hook of upper miniplates to distal hooks of lower miniplates at 10° to the occlusal plane (Fig. 3).
2. *In the 20° angulation model*, 250 g of load connects central hook of upper miniplates to the central hook of lower miniplates at 20° to the occlusal plane (Fig. 3).
3. *In the 30° angulation model,* 250 g of load connects the mesial hook of upper miniplates to the mesial hook of lower miniplates at 30° to the occlusal plane (Fig. 3).

### Post-processing stage

Stress distribution in the circummaxillary sutures and displacement of the surface landmarks in the maxillofacial bone were analyzed by using simulation software ANSYS software Version 12.1 (ANSYS Inc, Canonsburg, PA, USA). The database file from the HYPERMESH was transferred to the ANSYS software. Utilizing the software, nodal and element solutions were plotted, and areas of high stress concentrations were identified. Principle stresses and Von Mises stresses (in MPa) were measured separately in all simulations along with evaluation of displacement (in mm) of various craniofacial surface landmarks along the X, Y and Z coordinates. Superimpositions were created to depict the skeletal displacement as a result of altering the location and direction of force application. The stress values were exported to Microsoft Excel for analysis.

## Results

In the present study, intra-oral mechanics for skeletally anchored maxillary protraction (I-SAMP) were applied on two simulations (i.e. A and B between miniplates on maxilla and mandible on both right and left sides with three different angulations of force—10°, 20° and 30°). Three principle stresses, Von Mises stresses and displacement in all three axes of each finite element were outputed through stress nephrogram and displacement nephrogram respectively. Superimpositions to depict the skeletal displacement as a result of altering the location and direction of force application were studied thereafter.

### Stress distribution along the sutures

The patterns of stress distribution differed along the various sutures in all three dimensions of space. Even along the same suture, areas of tension and compression were evident. Maximum values of first, second and third principal stresses along with Von Mises stress were considered. Positive values of principle stresses depicted tensile stress, while negative value depicted the compressive stresses along the sutures.

**Fig. 4:**
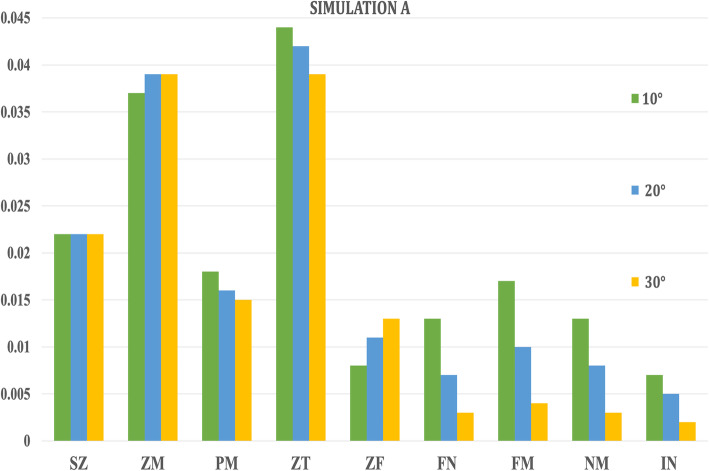
Von Mises stresses generated in craniofacial sutures with different angulations (10° to 30°) in Simulation A

**Fig. 5:**
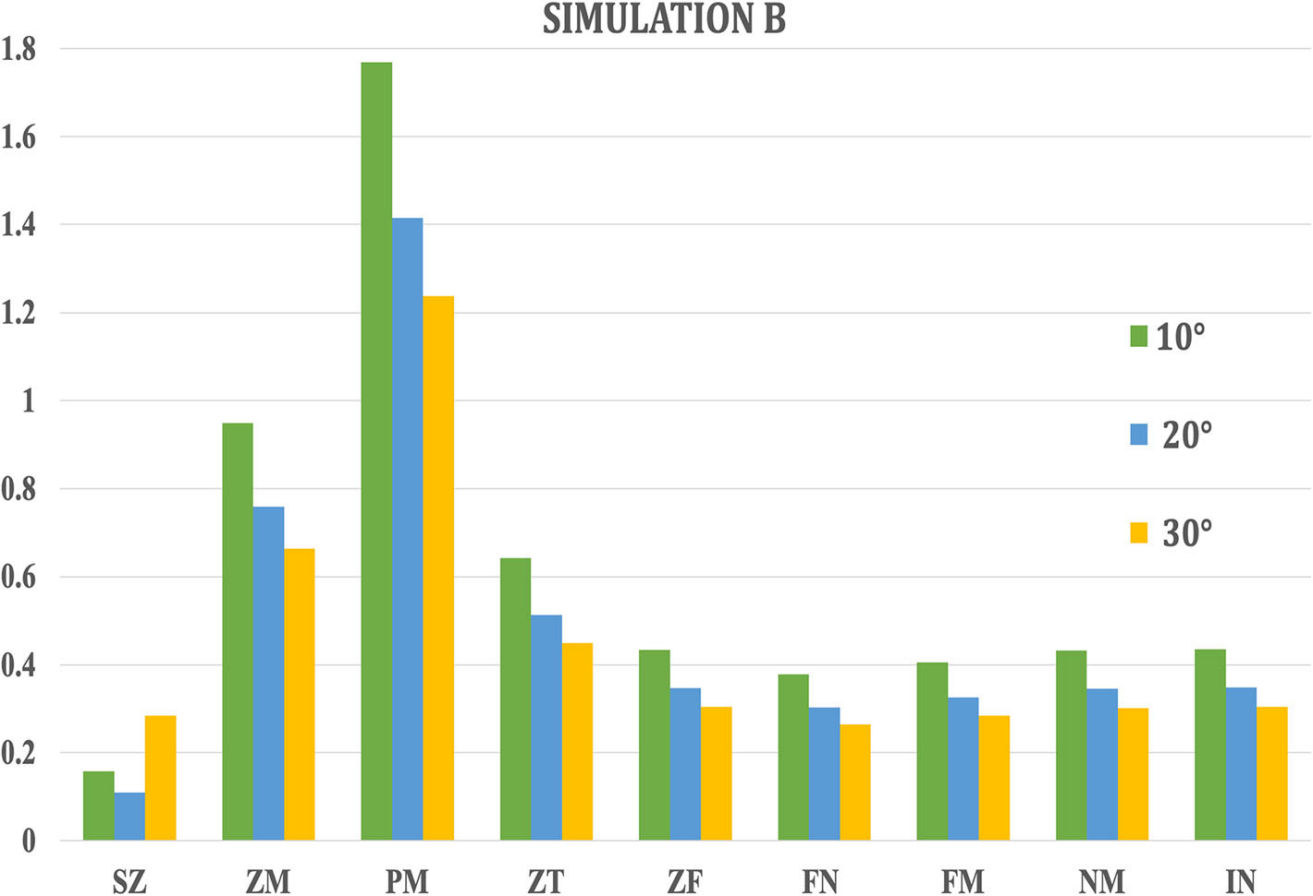
Von Mises stresses generated in craniofacial sutures with different angulations (10° to 30°) in Simulation B

In Simulation A, the highest amount of Von Misses stresses were witnessed in the zygomaticotemporal sutures (0.044 to 0.039 MPa) in all the three angulations of forces, while the lowest stresses were exhibited by the internasal (0.007 to 0.002 MPa) and frontonasal sutures (0.008 to 0.013 MPa) (Table 2, Fig. 4). On the other hand, in Simulation B, the highest amount of Von Misses stresses were seen in the pterygomaxillary sutures (1.768 to 1.238 MPa) in all the three angulations of forces and the lowest in the sphenozygomatic and frontonasal sutures (Table 2, Fig. 5).

**Table 2:** Stress pattern along the craniofacial sutures in maxillary protraction without maxillary expansion (Simulation A) and with expansion (Simulation B) when 250-g force was applied

	Angle of force 10° to occlusion	Angle of force 20° to occlusion	Angle of force 30° to occlusion
	SEQV	SEQV	SEQV
**Simulation A**			
**Sphenozygomatic suture (SZ)**	0.022	0.022	0.022
**Zygomaticomaxillary suture (ZM)**	0.037	0.039	0.039
**Pterygomaxillary suture (PM)**	0.018	0.016	0.015
**Zygomaticotemporal suture (ZT)**	0.044	0.042	0.039
**Zygomaticofrontal suture (ZF)**	0.008	0.011	0.013
**Frontonasal suture (FN)**	0.013	0.007	0.003
**Frontomaxillary suture (FM)**	0.017	0.010	0.004
**Nasomaxillary suture (NM)**	0.013	0.008	0.003
**Internasal suture (IN)**	0.007	0.005	0.002
**Simulation B**			
**Sphenozygomatic suture (SZ)**	0.158	0.109	0.284
**Zygomaticomaxillary suture (ZM)**	0.949	0.759	0.664
**Pterygomaxillary suture (PM)**	1.768	1.415	1.238
**Zygomaticotemporal suture (ZT)**	0.642	0.513	0.449
**Zygomaticofrontal suture (ZF)**	0.434	0.347	0.304
**Frontonasal suture (FN)**	0.379	0.303	0.265
**Frontomaxillary suture (FM)**	0.406	0.325	0.284
**Nasomaxillary suture (NM)**	0.432	0.346	0.302
**Internasal suture (IN)**	0.436	0.349	0.305

### Displacement pattern along the sutures

The highest forward displacement in Simulation A was exhibited by the pterygomaxillary suture closely followed by the zygomaticomaxillary suture. On increasing the angulations of force from 10° to 30°, there was a significant decrease in the displacement of the zygomaticomaxillary, pterygomaxillary, zygomaticotemporal and sphenozygomatic sutures along both the Y- and Z-axes.

A similar decrease in the displacement of the zygomaticomaxillary, pterygomaxillary, zygotemporal and sphenozygomatic sutures along both the Y- and Z-axes on increasing angulations of force from 10° to 30° was reported in Simulation B, although the highest forward displacement was seen in pterygomaxillary followed by the internasal suture.

### Displacement pattern of the maxillofacial bones

**Fig. 6:**
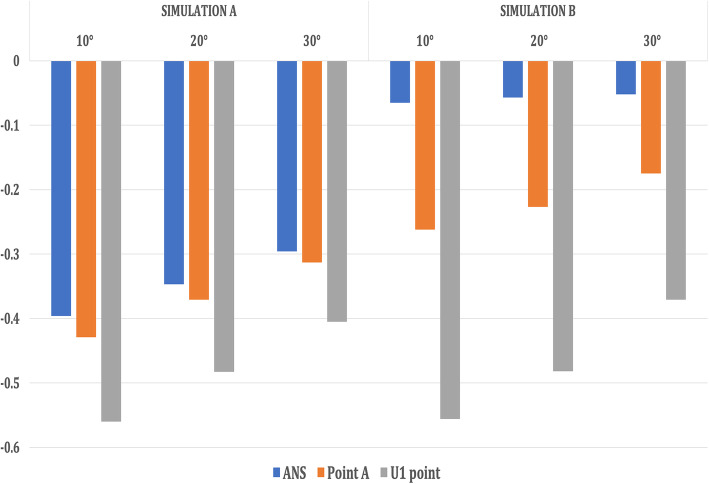
Antero-posterior displacement of the maxillary landmarks when 250 g of force was applied at the three angulations (10° to 30°)

**Fig. 7:**
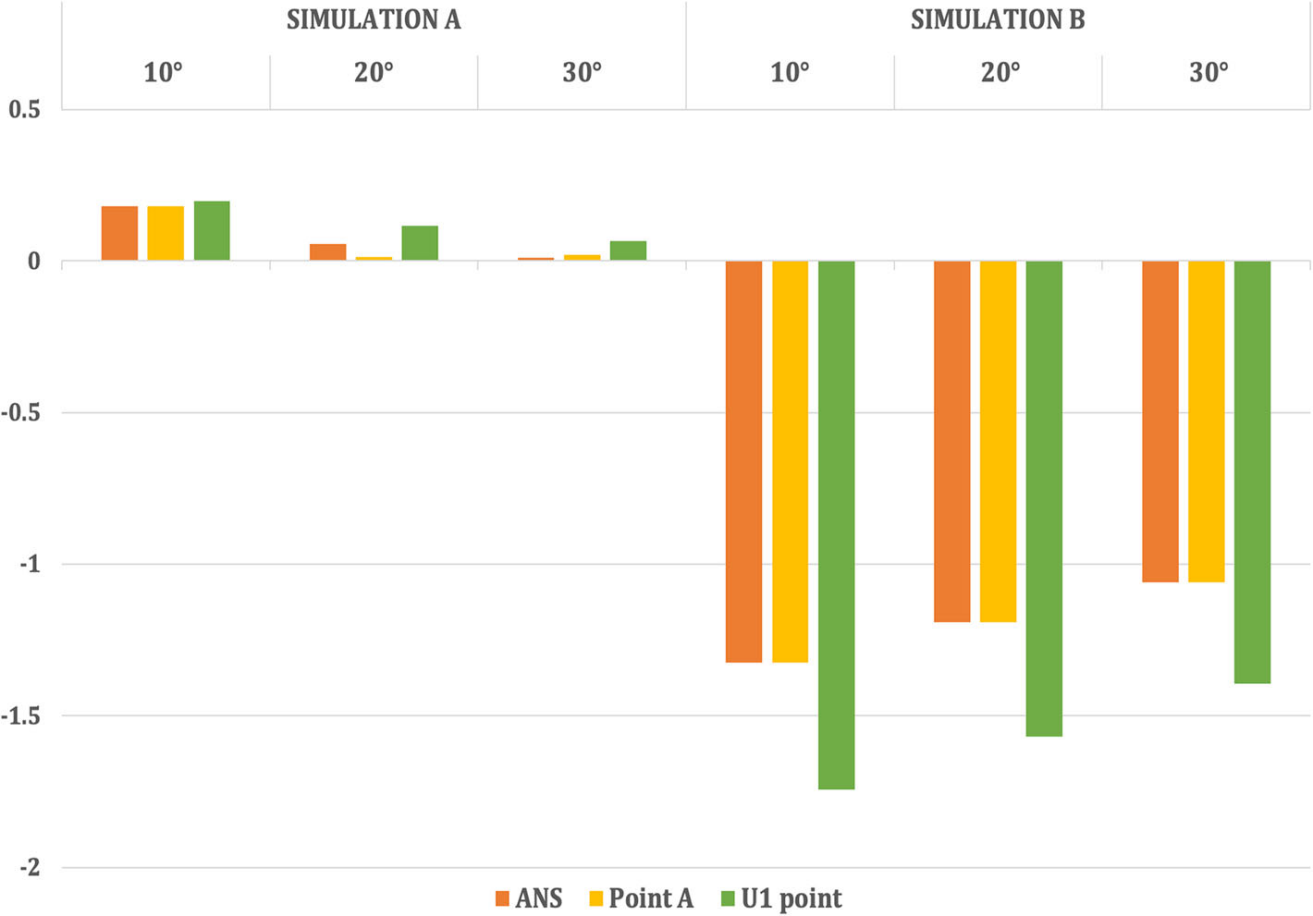
Vertical displacement of the maxillary landmarks when 250 g of force was applied at the three angulations (10° to 30°)

The entire maxilla moved upward and forward in an anti-clockwise direction, and the mandible underwent clockwise rotation leading to a backward displacement of Point B, Pogonion with a forward displacement of Condylion in Simulation A (Table 3, Figs. 6 and 7).

**Table 3:** Comparison of displacement of the surface landmarks in the maxillofacial structures with maxillary protraction without maxillary expansion (Simulation A) when 250-g force was applied

		Angle of force 10° to occlusion			Angle of force 20° to occlusion			Angle of force 30° to occlusion		
		X (mm)	Y (mm)	Z (mm)	X (mm)	Y (mm)	Z (mm)	X (mm)	Y (mm)	Z (mm)
**Simulation A**										
**Maxilla**	**ANS**	0.011	− 0.396	0.180	0.006	− 0.347	0.055	0.006	− 0.296	0.010
	**Point A**	0.011	− 0.429	0.180	0.006	− 0.371	0.012	0.006	− 0.313	0.020
	**U1 point**	0.018	− 0.560	0.196	0.013	− 0.483	0.115	0.012	− 0.405	0.065
**Mandible**	**Point B**	0.000	0.008	− 0.032	0.00	0.005	− 0.018	0.00	0.005	− 0.016
	**Pogonion**	0.000	0.017	− 0.032	− 0.001	0.010	− 0.018	0.00	0.009	− 0.016
	**Condylion**	− 0.002	− 0.007	0.004	0.002	− 0.003	0.002	0.002	− 0.003	0.001
**Simulation B**										
**Maxilla**	**ANS**	0.214	− 0.065	− 1.324	0.150	− 0.057	− 1.191	0.133	− 0.052	− 1.059
	**Point A**	− 0.134	− 0.262	− 1.324	− 0.116	− 0.227	− 1.191	− 0.089	− 0.175	− 1.059
	**U1 point**	− 1.674	− 0.556	− 1.743	− 1.506	− 0.482	− 1.569	− 1.399	-0.371	− 1.394
**Mandible**	**Point B**	0.000	0.004	− 0.008	0.000	0.002	− 0.008	0.000	0.002	− 0.007
	**Pogonion**	0.000	0.007	− 0.008	0.000	0.004	− 0.008	0.000	0.004	− 0.007
	**Condylion**	0.001	− 0.002	0.001	0.001	− 0.001	0.001	0.001	− 0.001	0.001

*X*, lateral displacement (+, lateral; −, median). *Y*, antero-posterior displacement (+, posteriorly; −, anteriorly). *Z*, Vertical displacement (+, superiorly; −, inferiorly).

U1 point—on the incisal edge of maxillary central incisor

L1 point—on the incisal edge of mandibular central incisor

**Fig. 8:**
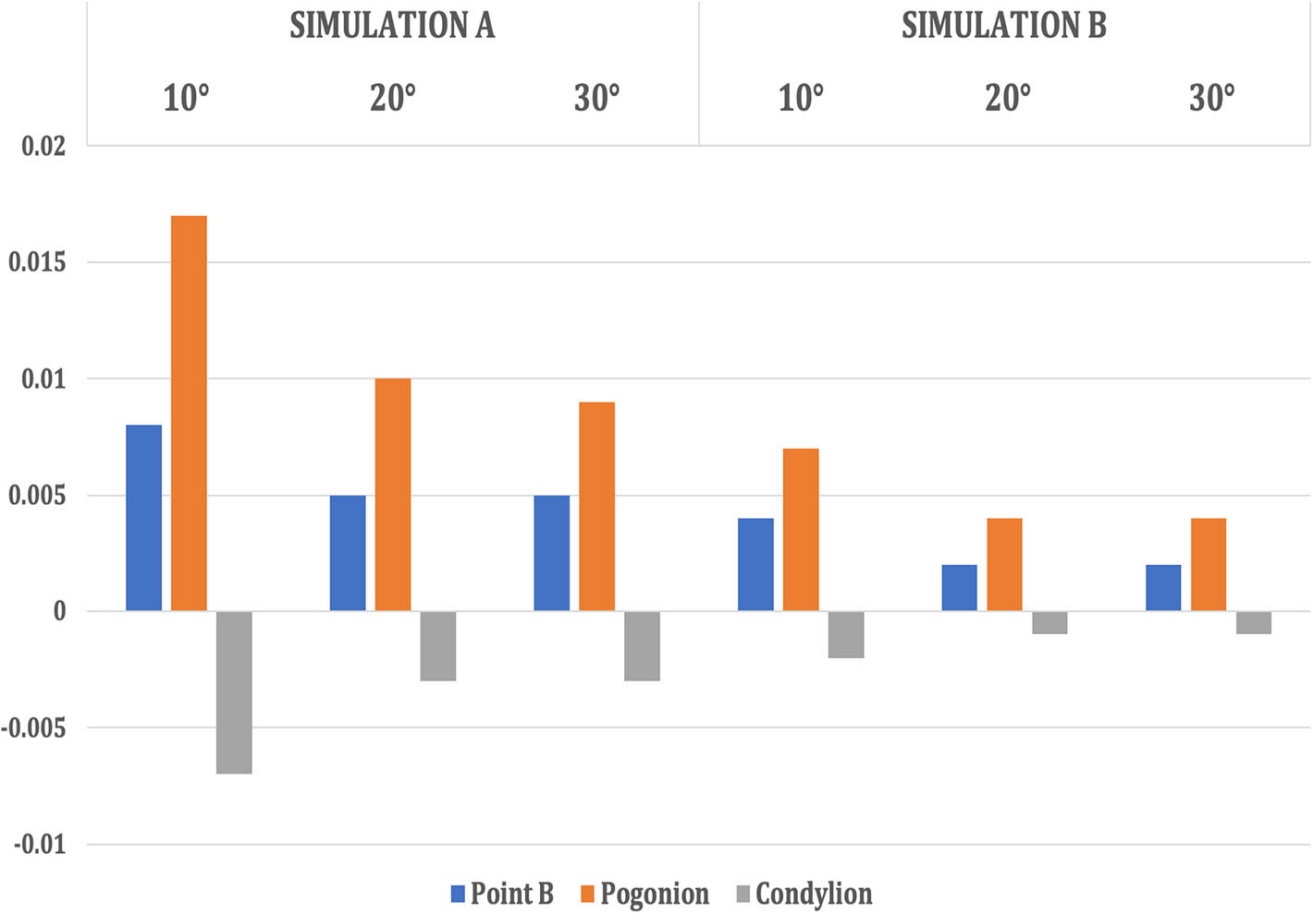
Antero-posterior displacement of the mandibular landmarks when 250 g of force was applied at the three angulations (10° to 30°)

**Fig. 9:**
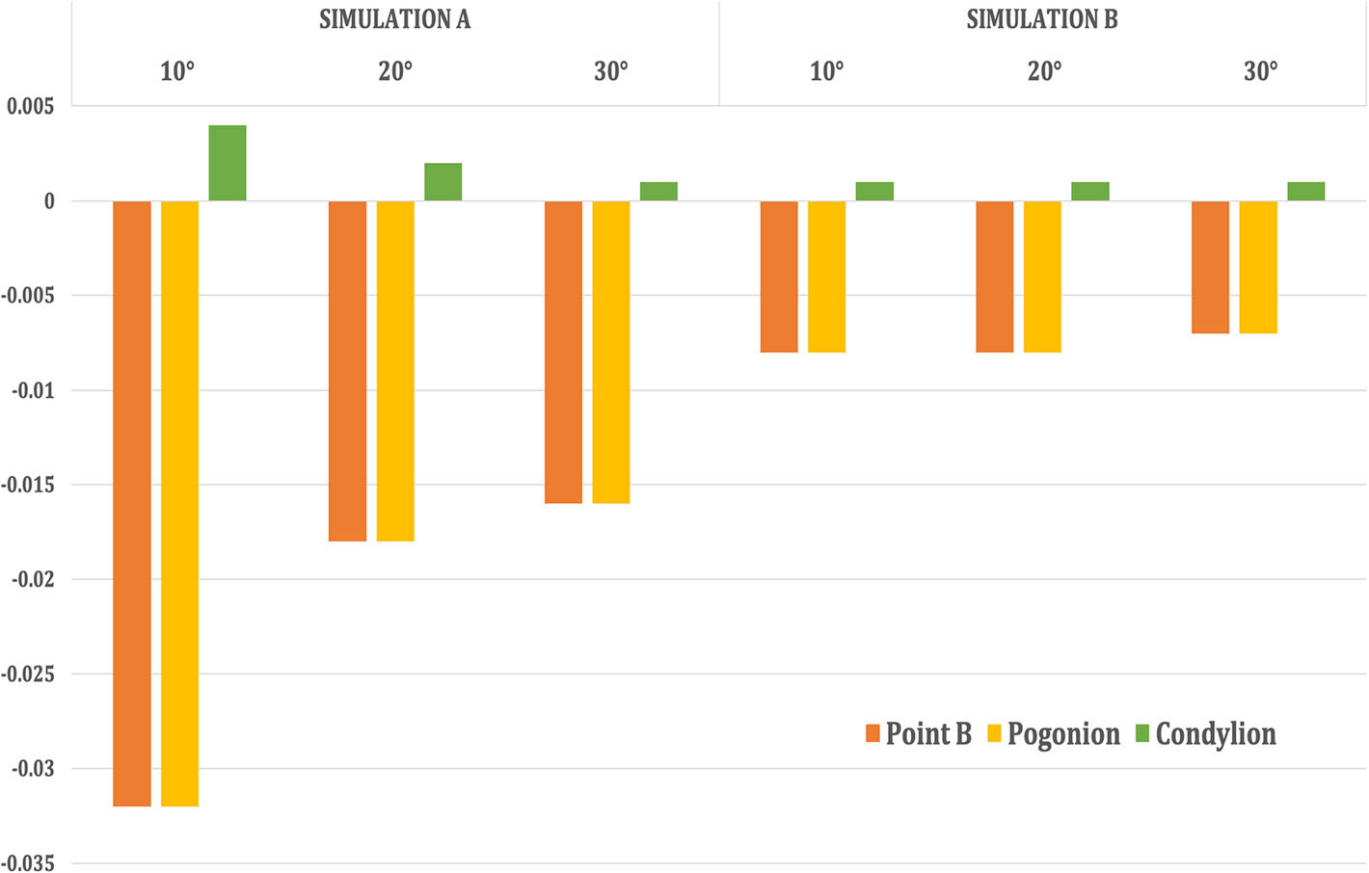
Vertical displacement of the mandibular landmarks when 250 g of force was applied at the three angulations (10° to 30°)

On the contrary, in Simulation B, the amount of maxillary translation was slightly more with rotation in a clockwise direction. However, the mandible underwent a similar clockwise rotation, leading to a backward displacement of Point B, Pogonion with a forward displacement of Condylion (Table 3, Figs. 8 and 9).

However, in both the simulations, as the angulations of force application increased, the forward displacement of maxilla decreased with lesser anti-clockwise and clockwise rotations of the maxilla respectively. In addition, the opening effect on the midpalatal suture also decreased.

### Stress distribution along the maxillofacial bones

The overall Von Mises stresses after maxillary protraction with midpalatal expansion were significantly higher than maxillary protraction without maxillary expansion with Class III intra-oral force. In the case of Simulation A, maximum stresses were seen in the maxilla around the site of screw placement for fixation of miniplates, while in simulation B, it existed from the anterior dentoalveolar region to the anterior nasal spine area in the maxilla (Table 4).

**Table 4:** Maximum Von Mises stresses in the maxillofacial structures when 250-g force was applied at three different angulations in two clinical simulations (A & B) (in MPa)

	***Simulation A (Maxillary protraction without midpalatal expansion)***		***Simulation B (Maxillary protraction with midpalatal expansion)***			
	10°	20°	30°	10°	20°	30°
**Nasal bone**	6.38	3.76	1.49	29.06	26.15	23.25
**Maxilla**	23.70	17.26	12.28	54.66	43.73	38.26
**Zygomatic bone**	21.47	21.83	21.85	28.26	22.89	20.04
**Temporal**	3.80	3.55	3.28	19.06	17.16	15.25
**Mandible**	6.65	2.96	1.55	7.82	6.25	5.00

### Superimpositions

Superimpositions of each simulation were created and colour-coded where the “before” image was shown in black mesh and the “after” image was displayed in a range of colours which directly correspond to the amount of Y displacement (pure protraction) or Z displacement (vertical) following force application. As the colour approached blue in the rainbow spectrum of colours, there was more anterior displacement in the Y-axis and downward displacement in the Z-axis of the skull model.

## Discussion

The treatment of Skeletal Class III malocclusion in patients continues to remain a challenge in orthodontic practice as orthodontics has evolved from an opinion-based practice into an evidence-based practice over the decades. Skeletal Class III malocclusion can arise subsequent to sole maxillary retrognathism, mandibular prognathism or a combination of the two [1].

Treatment of Skeletal Class III malocclusion due to maxillary retrognathism depends on multiple factors such the degree of skeletal correction desired, age of the patient, etc.. In growing individuals, correction by means of conventional methods such a facemask, chin or reverse twin block is preferable, while in adults, Class III warrants surgical correction [17]. However, the introduction of intra-oral tooth-borne anchorage by De Clerck et al. [4] and Cevidanes et al. [14] provides a different treatment modality for Class III correction in adolescents. Although multiple clinical studies for assessing the effects of intra-oral skeletally anchored Class III elastics (I-SAMP) are available, there is great variability in the results obtained. Thus, the aim of the present study was to analyse the stress distribution and displacement of maxillofacial structures in a Class III finite element model during maxillary protraction with intra-oral skeletally anchored Class III elastics (I-SAMP), to aid in improving clinical protocols.

The discussion has been divided into the following parts: finite element method and rapid maxillary expansion, displacement of various craniofacial structures with and without rapid maxillary expansion and stress pattern along the craniofacial sutures.

### Finite element method (FEM) and rapid maxillary expansion (RME)

The principle of the FEM is based on the division of a complex structure into smaller sections called elements where physical properties, such as the modulus of elasticity, are applied to indicate the object response against an external stimulus such as an orthodontic force [17].

Previous FEM studies by Moon W et al. [13] and Yan X et al. [18] have simulated clinical protocols where protraction forces were applied only on the maxilla using skeletal anchorage and have found that by varying the location and vector of Class III mechanics, orthodontists can differentially alter the magnitude of forward, downward and rotational movement of the maxilla. However, to the best of our knowledge, to this date, there is no FEM study evaluating the biomechanical effects on the maxillofacial complex of skeletally anchored Class III elastics with varying angulations.

The use of rapid maxillary expansion by conventional means or by the use of an Alt-RAMEC protocol has been advocated by Bacetti et al. [19] and Liou et al. [20] to attain greater maxillary advancement by distraction of the maxillary sutures. However, the benefits of expansion to aid in maxillary protraction continue to remain an enigma, as there is lack of substantial evidence suggesting any superior skeletal effects with expansion [21, 22]. Thus, the present study compares two simulations (A and B), without and with expansion to compare their effects on the craniofacial structures.

### Displacement of various craniofacial structures with and without rapid maxillary expansion

#### Craniomaxillary complex

In the present study, in Simulation A, the maxilla underwent forward displacement and counterclockwise rotation with the proclination of the maxillary anterior teeth irrespective of the angulations of load application, which were similar to the clinical findings, suggesting the reasonability and feasibility of the modeling. In Simulation B, the maxilla translated forward with clockwise rotation and proclination of the maxillary anterior teeth irrespective of the angulations of load application, which was again consistent with the clinical findings. However, the amount of anterior displacement of the surface landmarks of the maxilla was slightly more in Simulation B as compared with that in Simulation A, consistent with the findings of the previous study by Gautam et al. [23], who also advocated the use of maxillary expansion with protraction to attain augmented maxillary protraction. However, the FEM study by Jafari and Mohan [24] found little movement of the skull under expansion forces.

#### Mandibular fossa and mandible changes

In both Simulations A and B, the mandible underwent clockwise rotation leading to the backward displacement of Point B & Pogonion with the forward displacement of L1 point and Condylion. However, the amount of displacement in both antero-posterior and vertical directions was significantly higher in Simulation A than in B. The centre of rotation of the mandible seemed to be at Gonion as all values in the three axes were almost 0. These findings correspond to the findings of Morales-Fernandez et al. [25] in a systematic review, where they found that both skeletally and dentoalveolar-anchored dentofacial orthopaedics resulted in the clockwise rotation of the mandible and increase in inferio-anterior facial height clinically. However, antero-inferior displacement of the glenoid fossa was significantly higher in Simulation B in the present study.

### Effect of varying the angulations of Class III elastics

#### Craniomaxillary complex

With an increase in the angulation of force application from 10° to 30° in both Simulations A and B, the displacement of ANS, Point A and the U1 point decreased, portraying a decrease in the forward displacement of the maxilla. Additionally, the anti-clockwise and clockwise rotations of the maxilla in vertical direction and the opening effect on the midpalatal suture were decreased in Simulations A and B respectively.

#### Mandibular fossa and mandible changes

In the case of the mandible and glenoid fossa, in both the models, the mandible showed clockwise rotation, and the rotation degree decreased gradually with the increase of the angle from 10° to 30°. Similarly, antero-inferior displacement of the glenoid fossa also decreased with the increase of the angulation.

### Evaluation of stress distribution along the craniofacial sutures

The Von Mises stresses were used for this analysis because of the appropriateness, and the validity of the von Mises theory of failure [26]. In the present study, the magnitude of von Mises stress on the craniofacial sutures in Simulation B was hundred times of the stresses seen with Simulation A. The pattern of stress distribution and sutures experiencing maximum and minimum stresses also differed amongst the two simulations. The findings were similar to the study done by Gautam et al. [23] who also reported more overall principle and Von Mises stresses after maxillary protraction with maxillary expansion.

The present study reported that in Simulation B, the maximum stresses were seen in the pterygomaxillary suture followed by the zygomaticomaxillary, zygomaticotemporal and zygomaticofrontal sutures similar to the findings of Jafari et al. [24] and Gautam et al. [23] and in contrast to the findings of Ghomeima et al. [27]. The highest stresses were seen with the superior portions of the pterygomaxillary region compared with the inferior, similar to the findings of Gautam et al. [23], where these high stresses were responsible for the disarticulation of the palatal bone from the pterygoid process seen during rapid maxillary expansion, leading to further maxillary protraction.

Von Mises stresses in the internasal, maxillonasal, frontonasal and frontomaxillary sutures also increased with maxillary expansion and can be explained by the concept of Shetty et al. [28] which showed that anteriorly, the forces spread superomedially along the frontal process of the maxilla and the medial orbital wall up to the junction of the nasal and lacrimal bones. The present study also described the presence of differential strain patterns suggesting the possibility of differential bone remodelling along the same suture as documented previously by Oberheim and Mao [29] who showed contrasting bone strain patterns in the zygomatic arch across the zygomaticotemporal suture.

The absolute level of induced stresses greatly depends on bone elasticity and patient’s age. With the same orthopedic load, equivalent sutures of juvenile skulls experience significantly higher bone strain than adult skulls, suggesting that same mechanical force might have different biologic effects on immature and mature facial skeletons.

## Conclusion

Thus, it can be concluded based on the results of the finite element study, that I-SAMP with expansion is a desirable protocol for treatment of Class III patients having transverse and vertical deficiencies. The prescribed angulation of Class III elastics should be as low as possible, since the displacement and rotational effects on the craniomaxillary complex and mandible decreases with the increase in the angulation of elastics.

## Future scope

Although the amount of stresses and strains generated have been evaluated, the effects of multiple other factors such as the stage of skeletal maturation of the maxillofacial sutures, growth pattern of the individual and variation in the angulation of the elastics on the suture fibrogenesis and osteogenesis at a histological level are yet to be assessed and described. Moreover, the consequences of varying the magnitude of forces with different angulations on the temporomandibular joint complex need to be assessed to attain better clinical results.

## Data Availability

The datasets used and/or analysed during the current study are available from the corresponding author on reasonable request.
